# Relationship between deep subcutaneous abdominal adipose tissue and metabolic syndrome: a case control study

**DOI:** 10.1186/s13098-016-0127-7

**Published:** 2016-02-12

**Authors:** Se-Hong Kim, Ju-hye Chung, Sang-Wook Song, Won Sang Jung, Yun-Ah Lee, Ha-Na Kim

**Affiliations:** Department of Family Medicine, St. Vincent’s Hospital, College of Medicine, The Catholic University of Korea, 93-6 Ji-dong, Paldal-Gu, Suwon, Kyonggi-do 442-723 Republic of Korea; Department of Family Medicine, Uijeongbu St. Mary’s Hospital, College of Medicine, The Catholic University of Korea, 271, Cheon Bo-ro, Uijeongbu, Gyeonggi-do 480-717 Republic of Korea; Department of Radiology, College of Medicine, St. Vincent’s Hospital, College of Medicine, The Catholic University of Korea, 93-6 Ji-dong, Paldal-Gu, Suwon, Kyonggi-do 442-723 Republic of Korea

**Keywords:** Superficial subcutaneous adipose tissue, Deep subcutaneous adipose tissue, Visceral adipose tissue, Metabolic syndrome, Inflammatory cytokine, Adipocytokine

## Abstract

**Background:**

The deep subcutaneous adipose tissue (dSAT) is closely related to the obesity-associated complications similarly to the characteristics of visceral adipose tissue (VAT). However, the association between dSAT and metabolic syndrome (MS) is unclear. The purpose of our study was to evaluate the association of distinct abdominal adipose tissue with the cardiometabolic risk factors and MS.

**Methods:**

Abdominal computed tomography (CT) images were obtained in 365 asymptomatic subjects (187 subjects with MS and 178 without MS). The axial images segmented into superficial and deep SAT by manually tracing the fascia superficialis at L4–5 levels. The concentrations of serum inflammatory cytokines and adipokines were also measured.

**Results:**

The MS group had significantly lower adiponectin levels but significantly higher levels of resistin, leptin, tumor necrosis factor-alpha (TNF-α), interleukin-6 (IL-6), intercellular adhesion molecule (ICAM), monocyte chemotactic protein-1 (MCP-1), and oxLDL than the control group (*p* < 0.05). All inflammatory cytokines and adipokines were associated with the sum of VAT and dSAT areas (VDAT) (P for trend < 0.05), but no significant correlation was found between inflammatory cytokines and sSAT. dSAT was significantly associated with MS in both men and women (OR 2.371; *p* < 0.001) whereas the ORs between sSAT and MS were not significant (*p* = 0.597). The age-adjusted ORs between VDAT and MS (OR of 8.359 in men and 3.183 in women, *p* < 0.001) were higher than those of VAT (OR of 7.941 in men and 2.570 in women, *p* < 0.05) and dSAT (OR of 2.954 in men and 1.856 in women, *p* < 0.05).

**Conclusions:**

We demonstrated that dSAT was associated with increased inflammation and oxidative stress, suggesting that dSAT is an important determinant of MS. Therefore, abdominal subcutaneous fat should be considered as two functionally distinct compartments rather than a single entity.

## Background

Metabolic syndrome (MS) is a cluster of risk factors for type 2 diabetes and cardiovascular disease and is associated with increased mortality [1]. Although visceral adipose tissue (VAT) has been recognized as a key determinant of MS and is presumed to play an important role in its development [2, 3], the role of subcutaneous adipose tissue (SAT) is not well understood.

Abdominal SAT can be divided into superficial SAT (sSAT) and deep SAT (dSAT) by the fascial plane. dSAT has been reported to differ from sSAT morphologically and metabolically. Most of the dSAT is located in the posterior half of the abdomen, and the rate of lipolysis, lipogenesis, and inflammatory protein expression is higher in dSAT than in sSAT [4–6]. The fat lobules of these two subcompartments also differ in that sSAT is characterized by small tightly packed adipocytes organized in compact fascial septa, whereas the dSAT contains larger, less organized, and more vascularized lobules [7].

In general, dSAT is closely related to the pathophysiology of obesity complications in a manner nearly equivalent to the characteristics of VAT, while sSAT follows the pattern of lower-body SAT [8–10]. However, a debate has arisen regarding the relative contributions of dSAT compared with VAT in relation to MS. Although some previous studies suggested that the excessive deposit of dSAT results in abnormal lipid profiles and insulin resistance [11–13], the correlation of dSAT with cardiometabolic risk factors and MS was inconsistent [14, 15]. Therefore, the potential association between dSAT and the development of obesity-associated complications is still unclear. However, few studies have evaluated the distinct abdominal adipose tissue compartments associated with MS, cardiometabolic risk factors, and the expression of various cytokines.

The purpose of our study was 1) to examine the differences between sSAT and dSAT in relation to cardiometabolic risk factors and MS; 2) to determine the levels of various adipose tissue-derived cytokines in subjects with MS; 3) to evaluate the association of distinct abdominal adipose tissue compartments with the levels of inflammatory cytokines and adipokines.

## Methods

### Subjects

A total of 365 asymptomatic subjects (187 patients with MS and 178 subjects without MS) were recruited from the outpatient clinics at St. Paul’s Hospital, Uijeongbu St. Mary Hospital and St. Vincent’s Hospital in South Korea. All subjects received comprehensive health screening and underwent abdominal CT scans for screening purposes between September 2009 and March 2013. Subjects who underwent abdominal surgeries affecting the visceral fat distribution were excluded from the study. This study was approved by the Research Ethics Committee of the College of Medicine, The Catholic University of Korea, and written informed consent was obtained from all patients.

### Risk factor assessment and measurement of serum adipokine levels

The anthropometric, clinical and laboratory investigations were performed on all subjects. The height of each participant was determined using a fixed wall-scale measuring device and was measured to the nearest 0.1 cm. The body weight was measured to the nearest 0.1 kg using a digital scale calibrated prior to each measurement. The body mass index (BMI, kg/m^2^) was calculated as the weight in kilograms (kg) divided by the square of height in meters. Waist circumference (WC) was measured twice to the nearest centimeter at the end of normal expiration in a horizontal plane immediately superior to the left iliac crest according to the National Health and Nutrition Examination Survey protocols. If the variation between these two measurements was greater than 2 cm, a third measurement was taken and the mean was calculated using the two closest measurements. Seated blood pressures were measured using a mercury sphygmomanometer after a 10 min rest period. Two blood pressures measurements were taken from all subjects with a 5 min interval and were averaged for analysis.

Blood samples were drawn in the morning hours after a 12-h overnight fast. The samples were left at room temperature for 30 min, centrifuged for 15 min at 2500 rpm to separate the serum, and then stored at −70 °C. Fasting serum glucose, total cholesterol, triglyceride, and HDL-cholesterol were determined using an autoanalyzer (Hitachi 747 auto-analyzer, Hitachi, Tokyo, Japan). Insulin was measured by enzyme immunoassay (Immulite 2000, SIEMENS, IL, USA).

The concentrations of serum adiponectin, resistin, leptin, intercellular adhesion molecule (ICAM), monocyte chemotactic protein-1 (MCP-1), tumor necrosis factor-alpha (TNF-α), and interleukin-6 (IL-6) were measured in duplicate using enzyme-linked immunosorbent assay (ELISA) kits (Quantikine, R&D Systems, Minneapolis, MN, USA). The mean intra-assay and inter-assay coefficients of variation (CVs) of the adipokines and inflammatory cytokines analyzed were as follows: adiponectin (<4.7 %, <6.9 %), resistin (<5.3 %, <9.2 %), leptin (<3.3 %, 5.4 %), ICAM (<5.2 %, <7.8 %), MCP-1 (<7.8 %, <6.8 %), TNF-α (<5.2 %, 7.4 %), and IL-6 (<7.8 %, 9.6 %). A solid-phase two-site enzyme immunoassay (Mercodia Oxidized LDL ELISA, Mercodia, Uppsala, Sweden) was used to quantitatively measure oxidized low-density lipoproteins (oxLDL) in serum.

### Criteria for metabolic syndrome

MS was defined according to the revised NCEP-ATP III criteria with an ethnic-specific cutoff point for abdominal obesity [16]. Diagnosis of MS was based on the presence of three or more of the following clinical criteria: (1) WC ≥90 cm for men or ≥85 cm for women; (2) TG levels ≥150 mg/dl; (3) HDL-cholesterol levels <40 mg/dl for men or <50 mg/dl for women; (4) SBP ≥130 mmHg or DBP ≥85 mmHg, or the use of antihypertensive medication; and (5) FBS ≥100 mg/dl, or the use of anti-diabetics or insulin.

### Measurement of abdominal adipose tissue by CT

**Fig. 1:**
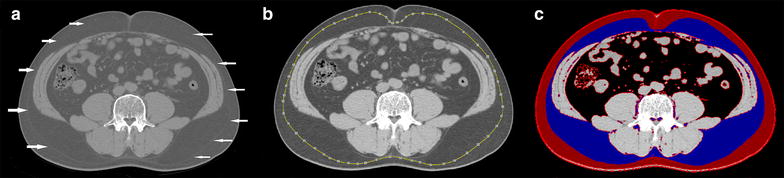
Measurement of deep and superficial subcutaneous adipose tissue area by cross-sectional abdominal computed tomography (CT) scans. The fascia superficialis (*arrowhead*) was used to separate superficial and deep compartments from SAT (**a**). The measurement of adipose tissue area was performed by tracing the fascia superficialis in the transverse CT image. After drawing the region of interest (ROI), the pixels with a threshold range of −190 to −30 Hounsfield units (HU) were identified as each adipose tissue area (**b**, **c**) as follows: *red color* superficial subcutaneous fat; *blue* deep subcutaneous fat; *black* visceral adipose tissue

To assess abdominal fat distribution, approximately 4–5 continuous transverse images (120 kV, 200 mA, scanning time of 2 s, field of view of 380 mm, and slice thickness 5 mm) were obtained at the level of the L4–5 intervertebral space using a CT scanner (LightSpeed, GE Healthcare, Milwaukee, WI). The cross-sectional areas of adipose tissue were measured by one experienced observer blinded to the clinical information of the study subjects. The segmentation of the axial images into superficial and deep SAT areas were performed by manually tracing the fascia superficialis at the L4–5 intervertebral space (Fig. 1). The pixels with a threshold range of −190 to −30 Hounsfield units (HU) were calculated for each area of adipose tissue. Visceral and deep subcutaneous adipose tissue (VDAT) area was calculated as the sum of VAT and dSAT areas. All CT analyses were performed using a dedicated offline workstation (Rapidia, software version 2.8, Infinitt, Seoul, Korea).

### Statistical analysis

Data were analyzed using SPSS version 21.0 (SPSS Inc., Chicago, IL) and presented as mean ± standard deviation (SD). The values of fasting plasma glucose, TG, and cytokines (adiponectin, resistin, leptin, ICAM, MCP-1, oxLDL, TNF-α, and IL-6) were highly skewed, thus, log-transformed for all analyses. Sex-specific, age-adjusted Pearson correlation coefficients were used to assess simple correlations between adipose tissue areas and the cardiometabolic risk factors. Analysis of covariance (ANCOVA) adjusted for gender and age was used to compare serum cytokine concentrations according to VDAT, VAT, dSAT, and sSAT tertiles. Multiple logistic regression analysis was performed to assess the relationships between each adipose tissue area and MS. Odds ratios (ORs) for MS were based on a 1-SD increase in each of the VDAT, VAT, dSAT, and sSAT areas. A two-sided *p* value <0.05 was considered statistically significant.

## Results

### Baseline characteristics of the study participants

Table 1 summarizes the baseline characteristics of the study participants. There was no significant difference in sex, age, and smoking status between the two groups. BMI (22.98 ± 3.84 kg/m^2^ vs. 27.24 ± 3.84 kg/m^2^, *p* < 0.001) and WC (82.29 ± 8.24 vs. 94.98 ± 6.82, *p* < 0.001) were significantly higher in the MS group than in the control group. Similarly, the VAT and SAT areas were greater in the MS group than in the control group (*p* < 0.05). The ratio of dSAT to total adipose tissue (TAT) area was higher in men than in women whereas the ratio of sSAT/TAT area was higher in women than in men (0.367 ± 0.097 vs. 0.245 ± 0.09, *p* < 0.001, data not shown). The MS group showed significantly higher glucose, TG, blood pressure, insulin, and HOMA-IR values and lower HDL cholesterol levels than the control group (*p* < 0.05). The MS group had significantly lower adiponectin levels (*p* < 0.001) but significantly higher levels of resistin, leptin, TNF-α, IL-6, ICAM, MCP-1, and oxLDL than the control group (*p* < 0.05).

**Table 1:** General characteristics of the study subjects (N = 365)

	Normal control (n = 178)	MS group (n = 187)	p value*
Male	91 (51.12 %)	107 (57.21 %)	0.250
Age (year)	51.208 ± 17.315	54.439 ± 14.344	0.054
Smoking status			0.341
Never/ex-smokers	139 (78.09 %)	156 (83.42 %)	
Current smokers	39 (21.91 %)	31 (16.58 %)	
Weight (kg)	61.595 ± 11.416	74.796 ± 14.067	<0.001
BMI (kg/m^2^)	22.985 ± 3.849	27.241 ± 3.841	<0.001
WC (cm)	82.296 ± 8.240	94.980 ± 6.821	<0.001
Glucose (mg/dL)	104.440 ± 38.161	114.934 ± 28.573	0.003
Triglyceride (mg/dL)	104.511 ± 56.979	187.348 ± 111.942	<0.001
HDL cholesterol (mg/dL)	53.836 ± 15.370	43.743 ± 11.334	<0.001
SBP (mm Hg)	119.483 ± 12.779	134.953 ± 16.053	<0.001
DBP (mm Hg)	71.730 ± 9.423	78.443 ± 13.344	<0.001
Insulin (µU/mL)	7.301 ± 6.730	11.677 ± 9.820	<0.001
HOMA-IR	1.883 ± 1.905	3.426 ± 3.405	<0.001
VAT (cm^2^)	78.041 ± 40.241	155.411 ± 52.241	<0.001
DSAT (cm^2^)	74.345 ± 34.487	113.896 ± 47.868	<0.001
SSAT (cm^2^)	80.891 ± 45.847	98.456 ± 48.189	0.001
SAT (cm^2^)	151.120 ± 73.331	211.384 ± 81.868	<0.001
Adiponectin (µg/mL)	6.306 ± 2.898	5.453 ± 5.228	<0.001
Resistin (ng/mL)	6.657 ± 4.006	8.614 ± 4.011	0.047
Leptin (ng/mL)	6.136 ± 6.474	6.656 ± 5.574	0.015
TNF-α (pg/mL)	3.402 ± 3.895	6.676 ± 5.707	0.003
IL-6 (pg/mL)	2.613 ± 1.995	3.306 ± 1.532	0.004
ICAM (ng/mL)	118.062 ± 48.877	166.617 ± 65.287	<0.001
MCP-1 (pg/mL)	245.731 ± 80.592	304.719 ± 92.347	<0.001
oxLDL (U/L)	65.043 ± 16.715	85.536 ± 21.876	<0.001
L/A	1.301 ± 1.755	1.739 ± 1.918	0.024

Values are expressed as the mean ± SD (standard deviation), *n* number of subjects, *BMI* body mass index, *WC* waist circumference, *SBP* systolic blood pressure, *DBP* diastolic blood pressure, *VAT* visceral adipose tissue, *DSAT* deep subcutaneous adipose tissue, *SSAT* superficial subcutaneous adipose tissue, *TNF-α* tumor necrosis factor-alpha, *IL-6* interleukin-6, *ICAM* intercellular adhesion molecule, *MCP-1* monocyte chemoattractant protein 1, *oxLDL* oxidized low-density lipoprotein, *L/A* leptin to adiponectin ratio

* Statistical significance was tested using independent t-tests or χ^2^ test

### Correlations with cardiometabolic risk factors

The correlations between adipose tissue area and cardiometabolic risk factors are shown in Table 2. All cardiometabolic risk factors were significantly correlated with VAT except for diastolic blood pressure in men. The dSAT area in men and women had similarly high correlations with most of the cardiometabolic risk factors. Waist circumference (WC) was significantly correlated with all subdivisions of abdominal adiposity but was more strongly associated with VAT and dSAT than with sSAT. The correlation coefficients of fasting blood glucose (*r* = 0.489, *p* < 0.001), TG (*r* = 0.553, *p* < 0.001), HDL (*r* = −0.579, *p* < 0.001), and systolic blood pressure (SBP) (*r* = 0.316, *p* < 0.01) with VAT were higher than those with dSAT in men (*r* = 0.351, 0.209, −0.321, and 0.306, respectively, *p* < 0.05). Glucose and SBP were also correlated with the area of sSAT in men, but weak in comparison to dSAT. We did not find significant correlations of fasting blood glucose, TG, and HDL with dSAT and sSAT in women.

**Table 2:** Partial correlation coefficients between metabolic risk factors and adipose tissue areas (age-adjusted)

	Male			Female		
	VAT	dSAT	sSAT	VAT	dSAT	sSAT
WC (cm)	0.732*	0.753*	0.549*	0.700*	0.634*	0.623*
Glucose (mg/dL)	0.489*	0.351**	0.246***	0.386**	0.191	0.119
Triglyceride (mg/dL)	0.553*	0.209***	0.271***	0.497***	0.063	0.135
HDL cholesterol (mg/dL)	−0.579*	−0.321**	−0.138	−0.485***	−0.222	−0.130
SBP (mmHg)	0.316**	0.306**	0.214***	0.340**	0.464*	0.317**
DBP (mmHg)	0.146	0.095	0.206	0.302**	0.381*	0.249***
Insulin	0.307**	0.374*	0.415*	0.517*	0.241***	0.281***
HOMA-IR	0.360**	0.404*	0.427*	0.536*	0.251***	0.280***

*VAT* visceral adipose tissue, *DSAT* deep subcutaneous adipose tissue, *SSAT* superficial subcutaneous adipose tissue, *WC* waist circumference, *SBP* systolic blood pressure, *DBP* diastolic blood pressure, *HOMA-IR* homeostasis model assessment of insulin resistance

* *p* < 0.001, ** *p* < 0.01, *** *p* < 0.05

### Comparison of the cytokine levels according to abdominal adiposity

**Fig. 2:**
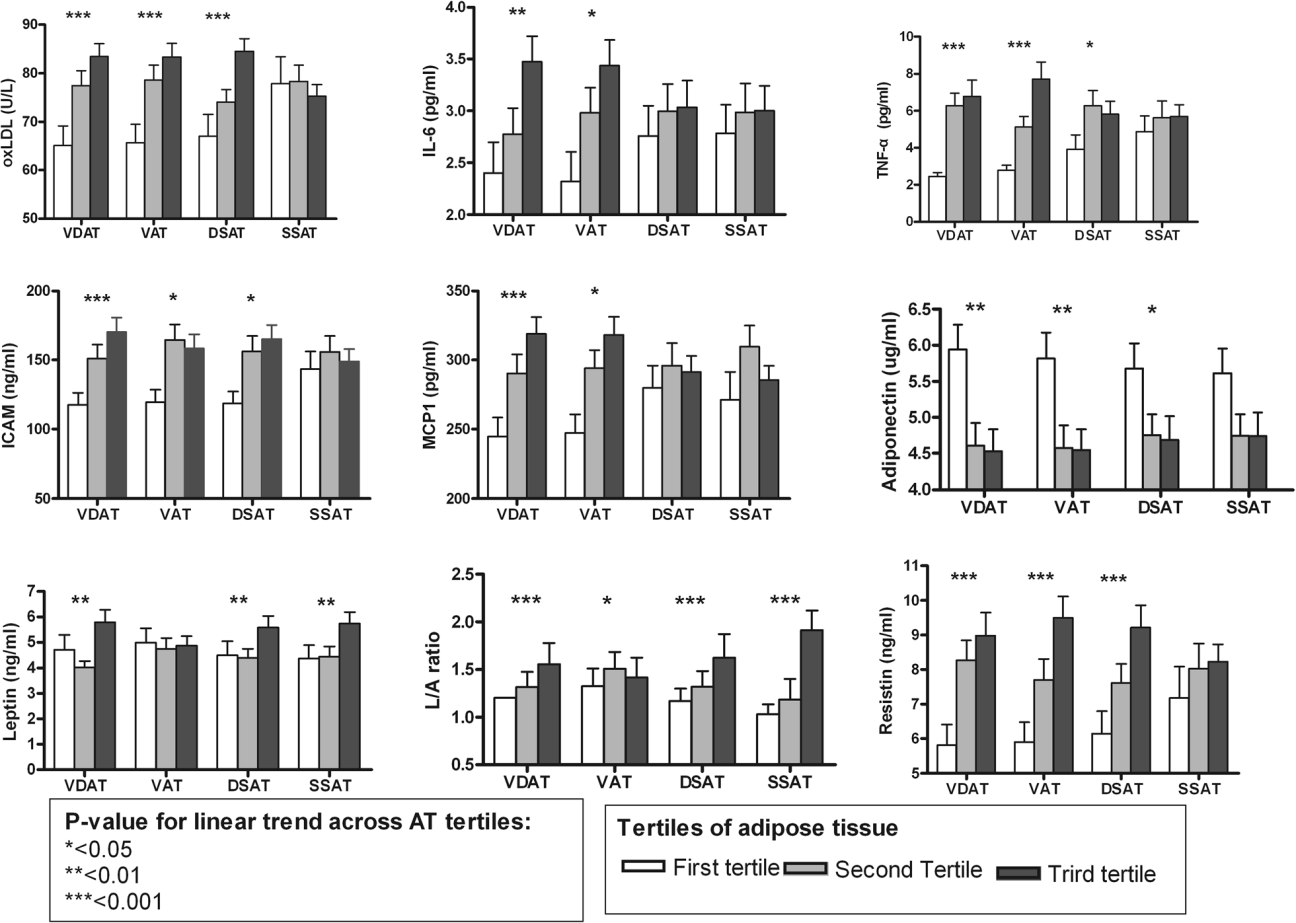
Comparison of the cytokine levels according to the subdivisions of abdominal adiposity in male. The areas of visceral and deep subcutaneous adipose tissue (VDAT), visceral adipose tissue (VAT), deep subcutaneous adipose tissue (dSAT), and superficial subcutaneous adipose tissue (sSAT) were divided into tertiles for comparison of various cytokines. Values are expressed as mean ± standard error of the mean

**Fig. 3:**
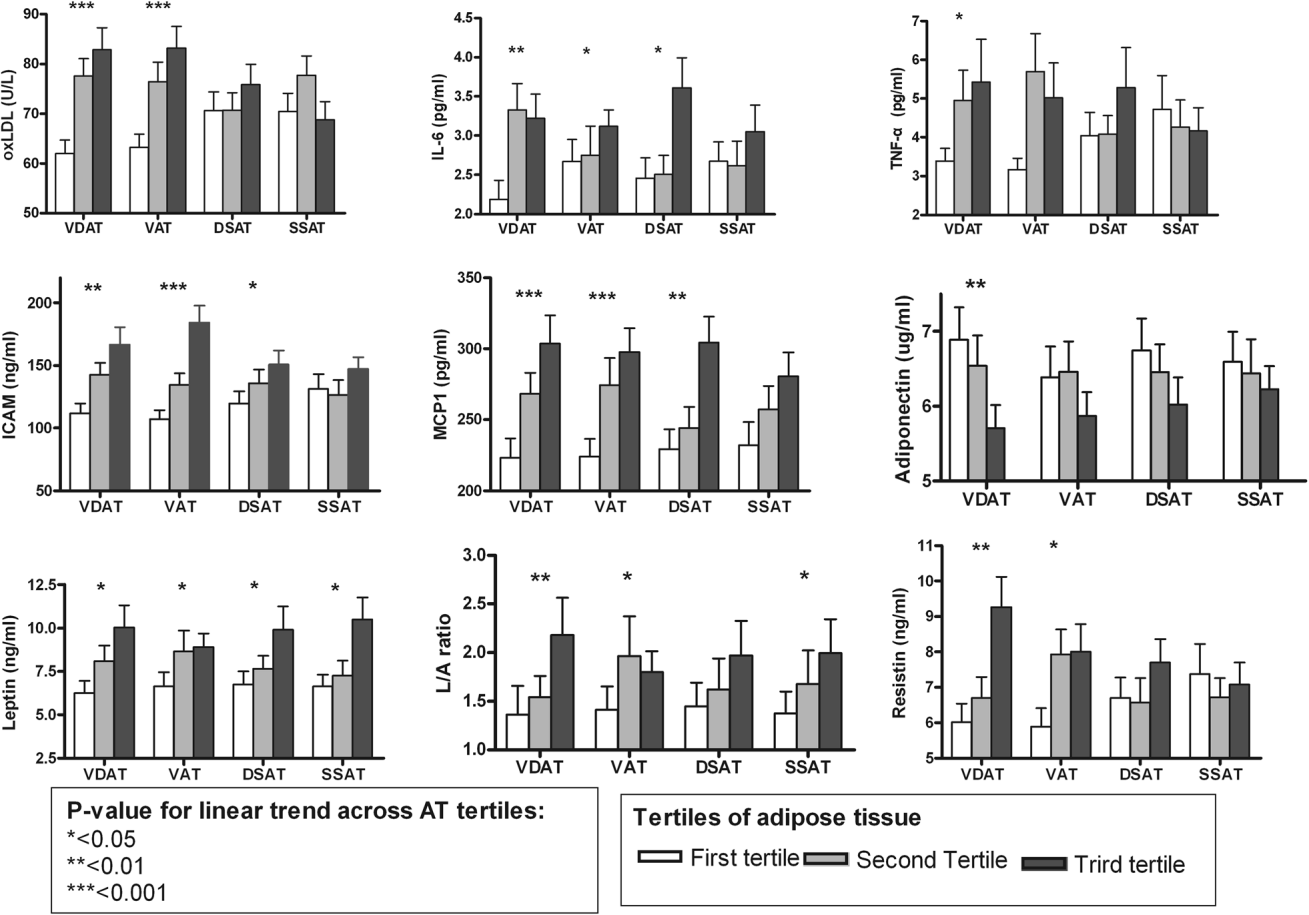
Comparison of the cytokine levels according to the subdivisions of abdominal adiposity in female. The areas of visceral and deep subcutaneous adipose tissue (VDAT), visceral adipose tissue (VAT), deep subcutaneous adipose tissue (dSAT), and superficial subcutaneous adipose tissue (sSAT) were divided into tertiles for comparison of various cytokines. Values are expressed as mean ± standard error of the mean

We divided subjects into tertiles according to the subdivisions of abdominal adiposity. All inflammatory cytokines were associated with increasing VDAT tertile, but no significant correlation was found between inflammatory cytokines and sSAT in males and females (*p* for trend <0.05, Figs. 2, 3). In male, significant associations were observed between dSAT tertiles and most of the inflammatory cytokines and adipokines (*p* < 0.05, Fig. 2). Leptin, L/A ratio, and resistin increased linearly with each increase in dSAT tertile: leptin levels were 4.493 ± 4.46, 4.392 ± 2.73, and 5.577 ± 3.72 for the first, second, and third dSAT tertiles, respectively (*p* = 0.002); resistin levels for dSAT tertiles were 6.149 ± 3.49, 7.618 ± 3.24, and 9.213 ± 4.40, respectively (*p* < 0.001). In addition, the serum level of adiponectin decreased with each tertile increase in VAT and dSAT: adiponectin for each dSAT tertile were 5.679 ± 2.76, 4.755 ± 2.21, and 4.683 ± 2.70, respectively (*p* = 0.032). Similarly, inflammatory cytokine concentrations increased with each increase in dSAT tertile except for IL-6 and MCP-1.

In female, Leptin, IL-6, ICAM and MCP-1 increased linearly with each increase in dSAT tertile (*p* < 0.05, Fig. 3). While the serum level of adiponectin decreased with increase in dSAT tertile, no significant differences were found between the tertiles: adiponectin for each dSAT tertile were 6.74 ± 3.12, 6.454 ± 2.63, and 6.02 ± 2.67, respectively (*p* = 0.643). Most of inflammatory cytokine concentrations increased with each increase in VAT tertile except for TNF-α.

### Association between abdominal adipose tissue subcompartments and MS

The association between each adipose tissue area and MS was assessed using multivariate logistic regression models (Table 3). dSAT was significantly associated with MS in both men and women (OR 2.371; 95 % CI 1.563–3.594; *p* < 0.001) whereas the ORs between sSAT and MS were not significant (*p* = 0.597). The age-adjusted ORs between VDAT and MS (OR of 8.359 in men and 3.183 in women, *p* < 0.001) were higher than those of VAT (OR of 7.941 in men and 2.570 in women, *p* < 0.05) and dSAT (OR of 2.954 in men and 1.856 in women, *p* < 0.05).

**Table 3:** Multiple logistic regression analysis of metabolic syndrome

	B	SE	OR	90 % CI	p value
Total					
VDAT	1.628	0.184	5.094	3.55–7.31	<0.001
VAT	1.568	0.202	4.795	3.228–7.124	<0.001
dSAT	0.863	0.212	2.371	1.563–3.594	<0.001
sSAT	0.098	0.186	1.103	0.766–1.588	0.597
Male					
VDAT	2.123	0.306	8.359	4.591–15.219	<0.001
VAT	2.072	0.350	7.941	3.998–15.771	<0.001
dSAT	1.083	0.376	2.954	1.413–6.178	0.004
sSAT	0.054	0.384	1.055	0.497–2.240	0.888
Female					
VDAT	1.158	0.231	3.183	2.023–5.008	<0.001
VAT	0.944	0.272	2.570	1.508–4.380	0.001
dSAT	0.618	0.264	1.856	1.107–3.111	0.019
sSAT	−0.103	0.271	0.902	0.531–1.534	0.705

Age-adjusted odds ratios (ORs) and 95 % confidence intervals (CIs) are presented as odds for metabolic syndrome with 1 SD increase in abdominal adipose tissue areas

*VDAT* the sum of visceral and deep subcutaneous adipose tissue, *VAT* visceral adipose tissue, *dSAT* deep subcutaneous adipose tissue, s*SAT* superficial subcutaneous adipose tissue

## Discussion

In this study, dSAT as well as VAT was associated with MS in both men and women. The CT measurements of dSAT were well correlated with multiple metabolic risk factors, and these risk factors were more strongly correlated with dSAT than with sSAT. In addition, a significant association was observed between dSAT and most of the inflammatory cytokines and adipocytokines but no significant correlations were found with sSAT. Moreover, dSAT was significantly associated with MS in both men and women but the ORs between sSAT and MS were not significant. These results suggest that dSAT may contribute to the obesity-related complications in a nearly same pattern to that observed for VAT.

Many previous studies have demonstrated that VAT is strongly associated with cardiometabolic risk factors and MS. However, there is a controversy as to whether VAT alone is responsible for the metabolic complications due to obesity. Although several investigators have reported that SAT may also contribute to MS and insulin resistance [10, 17–19], the correlation between SAT and MS was inconsistent. This inconsistency may result from the study of metabolically unhealthy dSAT. However, only a few studies have evaluated the cardiometabolic risk of dSAT so far, and the results were inconsistent according to the study populations. Some investigators have reported a significant association between dSAT and insulin sensitivity [6, 9, 12], non-alcoholic steatohepatitis [20], and adverse lipid and glycemic profiles [8, 11]. In contrast, other studies found no correlation of dSAT with postprandial TG and lipid profile in patients with coronary artery disease or type 2 diabetes mellitus [14, 15]. This conflicting result might be due to differences in sample size and inclusion criteria. Our study demonstrated that dSAT as well as VAT were associated with MS, and showed a strong correlation with most metabolic risk factors compared with sSAT. Indeed, dSAT was significantly associated with MS in both men and women, whereas the ORs between sSAT and MS were not significant (*p* = 0.597). Furthermore, the age-adjusted ORs between VDAT and MS were higher than those of VAT or dSAT, and all inflammatory cytokines were also associated with increasing VDAT tertile. These findings suggest that the sum of VAT and dSAT rather than VAT alone would be a better predictor for metabolic complication that is not completely explained by VAT or dSAT.

In this study, ORs of dSAT for MS and the correlations between dSAT and metabolic risk factors were higher in men than in women. Furthermore, the association between dSAT tertiles and the inflammatory cytokines was more apparent in men than in women. Such findings are similar to that of a previous study, which reported that dSAT was more weakly associated with health risks in women compared with men [9, 21, 22]. However, the reasons for the more strong association of dSAT with various cytokines and MS in men remain unclear; the relatively small sample size of women compared to men in this study might not fully explain this finding. The cause of these sex differences may be related to a twofold higher rate of free fatty acid mobilization from fat cells by norepinephrine stimulation in men compared with women [23]. Another possible explanation is the difference in sexually dimorphic subcutaneous fat distribution. Consistent with previous study [8, 9], the sSAT to TAT ratio in the present study was higher in women than in men, and the relatively large amount of sSAT in women might attenuate the influence of VAT and dSAT on the association with MS and cytokines in women as compared to men. Considering ethnic difference of fat distribution, further studies are need to clarify whether our findings in sex differences are limited to Korean population or generalized to Asian populations.

It is well known that adipose tissue produces various cytokines, such as resistin, leptin, adiponectin, IL-6, TNF-α, and MCP-1. The excessive secretion of inflammatory cytokines and decreased secretion of defensive adipocytokines, such as adiponectin, may cause obesity-related chronic or low-grade systemic inflammation [24]. The MS group had significantly lower adiponectin levels but significantly higher levels of resistin, leptin, TNF-α, IL-6, ICAM, MCP-1, and oxLDL compared with the control group. Moreover, most cytokines were similarly associated with VAT and dSAT but not with sSAT. This trend could be explained by metabolically active deep subcutaneous adipocytes. It has been well established that inflammatory, lipogenic, and lipolytic genes are overexpressed in dSAT [7], reflecting the protein expression characteristics of VAT [4, 6]. In addition, the percentage of small adipocytes and saturated fatty acids increases in dSAT [7, 25], which indicates decreased fat storage capacity, leading to excessive inflammation [26]. To the best of our knowledge, this is the first study in which various cytokines have all been investigated in relation to SAT subcompartment distribution and MS.

Although adiponectin is considered an important modulator of MS to overt atherosclerosis [27], little is known about the relationship between adiponectin and dSAT. Some previous studies found no correlation between plasma adiponectin levels and SAT [28, 29]. On the contrary, other studies showed an inverse association between adiponectin and SAT [17, 30, 31]. These inconsistent findings may result from methodological limitations of the measurement of adipose tissue area using CT, combining two different types of SAT into a single entity. In our study, adiponectin levels were negatively associated with VAT and dSAT areas but not associated with sSAT, more pronounced for men. In contrast, serum leptin level was positively associated with both SAT subcompartments, and L/A ratio was correlated with most of adipose tissue parameters. The result of our study implies that adiponectin is a more specific biomarker for metabolically unhealthy obese (MUHO) than leptin and L/A ratio. Regarding the gender difference, our results are in line with the previous studies [21, 22], and explained by the predominant dSAT deposition and the lower expression of adiponectin in male compared to female subjects. Contrary to our results, one recent study on SAT subcompartments had demonstrated sSAT-specific downregulation of adiponectin and increase of inflammatory cytokines [31]. However, this study evaluated the expression of proteins from adipose tissue with the relatively small number of subjects. Considering the differences between circulating level and the adipose tissue expression of cytokines, further studies are required to clarify the role of sSAT on metabolic complication.

In this study, serum TNF-α concentration was similarly associated with VAT and dSAT in male, but not in female. Consistent with our findings, Koistinen et al. reported that subcutaneous adipose tissue TNF-α mRNA level correlated with BMI in men but not in women [32]. However, the systemic release of TNF-α is variable, thus abdominal adiposity may not influence peripheral TNF-α concentrations [33, 34]. For oxLDL and resistin, the serum levels increased in the MS group compared with the control group. In addition, oxLDL and resistin levels increased with each increase in VAT and dSAT tertile in male. In contrast to this result, some previous studies had demonstrated that VAT is correlated with plasma oxLDL level but not with SAT [35, 36]. In addition, the relationship between resistin levels and abdominal SAT distribution has been also inconsistent and unclear. Although Utzschneider et al. reported a correlation between resistin levels and BMI and SAT [37], other studies found no association between resistin levels and obesity [38, 39] or SAT [40]. However, these studies measured abdominal fat depositions only in small subjects and did not separate SAT into superficial and deep compartments. Considering the metabolic differences between dSAT and sSAT, our study clearly indicates that dSAT as well as VAT may play an important role in systemic oxidative stress and MS.

There are some limitations in this study. First, our findings may not be applicable to the general population because of the relatively small sample size. Furthermore, we did not take into account the levels of physical activity and menopausal state, both of which may affect visceral adiposity. Second, we performed a cross-sectional study, and could not determine causality between dSAT and MS. Third, although total adiponectin levels were negatively associated with VAT and dSAT, we did not measure the high molecular weight (HMW) adiponectin, the active forms of adiponectin. Fourth, we measured AT area in a single cross-sectional image rather than AT volume. Although multislice volume imaging is generally considered gold standard for measuring adipose tissue volumes, its application is limited by radiation exposure associated with multislice CT. Accordingly, most investigators use a single cross-sectional image at the level of L4–5 intervertebral space to assess abdominal adiposity, which is known to well correlate with AT volume. Moreover, our study design has strength in allowing for an exact analysis of abdominal SAT distribution by anatomical landmark, providing robust evidence for measuring dSAT to assess cardiometabolic risk.

## Conclusions

In this study, we demonstrated that dSAT was associated with MS, increased inflammation, and oxidative stress, suggesting that dSAT is an important determinant of MS. Therefore, from the perspective of early effective intervention for MS, abdominal subcutaneous fat should be considered as two functionally distinct compartments rather than a single entity. Further prospective studies are required to determine the correlations between these compartments and metabolic risk factors over time in order to evaluate the influence of dSAT on cardiometabolic risk.
